# Earthworms (Oligochaeta: Lumbricidae) of Interior Alaska

**DOI:** 10.3897/BDJ.6.e27427

**Published:** 2018-07-10

**Authors:** Megan Booysen, Derek Sikes, Matthew L. Bowser, Robin Andrews

**Affiliations:** 1 West Valley High School, Fairbanks, United States of America; 2 University of Alaska Museum, Fairbanks, United States of America; 3 U.S. Fish & Wildlife Service, Kenai National Wildlife Refuge, Soldotna, United States of America; 4 Department of Biology and Wildlife, University of Alaska Fairbanks, Fairbanks, United States of America

**Keywords:** Clitellata, Megadrili

## Abstract

Earthworms in the family Lumbricidae in Alaska, which are known from coastal regions, primarily in south-central and south-eastern Alaska, are thought to be entirely non-native and have been shown to negatively impact previously earthworm-free ecosystems in study regions outside of Alaska. Despite occasional collections by curious citizens, there had not been a standardised earthworm survey performed in Interior Alaska and no published records exist of earthworms species from this region. Mustard extraction was used to sample six locations that differed in elevation, mostly in the College region of Fairbanks, Alaska. Two of the six locations yielded earthworms. There was no relationship between earthworm abundance and elevation (*p* = 0.087), although our sample size was small. Our sampling, combined with specimens in the University of Alaska Museum, has documented four exotic species and one presumed native species of lumbricid earthworms in Interior Alaska.

## Introduction

Most earthworms found in previously glaciated areas of North America are thought to be invasive (Bohlen et al. 2004). Although Interior Alaska (north of the Alaska Range and south of the Brooks Range) was not glaciated during the Pleistocene (Behan 1978, Pielou 1991, Shafer et al. 2010), a lack of historical observations suggests Interior Alaska is without native lumbricid earthworms either due to failure to establish populations or extinction due to cold and dry soil conditions prevalent during the last 2.6 million years (Reynolds 1995, Tiunov et al. 2006, Shafer et al. 2010). However, it is still possible that native lumbricid earthworms could be found in historically unglaciated parts of Interior Alaska. *Bimastos parvus* (Eisen, 1874) has been found in a glacial refugium in the Yukon Territory, where it is believed to be native (Berman and Marusik 1994, Reynolds 2015).

Human activity has been the primary method for introduction of peregrine European and Asian earthworms into previously earthworm-free ecosystems (Blakemore 2009). Earthworms and their egg-filled cocoons can be spread when packed into tyre treads or dumped at recreational sites where they are used as bait, transported in plant soils and via waterways, added intentionally to gardens or discarded in local woods after people have finished using them as vermicomposters (Cameron et al. 2007, Rogers and Collins 2017). However, the common vermicomposting species, *Eisenia fetida* (Savigny, 1826), apparently cannot survive outside of compost bins in south-central Alaska (Bowser 2015) or presumably northwards. As a result of presumed human introductions, Alaska now has approximately 14 species of feral lumbricid earthworms which, until recently, were known only from coastal Alaska, primarily in the state's south-central and south-eastern regions (Bowser 2015, Reynolds 2016, Saltmarsh et al. 2016). Six species of lumbricids are reported from Yukon Territory by Reynolds (2015), but no published records exist for lumbricids in Interior Alaska.

Non-native earthworms' dramatic negative impacts on previously earthworm-free ecosystems have been well documented in temperate and boreal landscapes (Hale et al. 2006, Sackett et al. 2012, Rogers and Collins 2017) but have not yet been studied in Alaska. Due to the loss of forest floor organic layers and negative impacts on seed banks, exotic earthworms have been shown to lower native plant diversity (Hale et al. 2006, Hopfensperger et al. 2011, Craven et al. 2016). It is thought that the feeding habits of earthworms allow for non-native plants to establish when fungal-plant root symbioses are altered (Bohlen et al. 2004). This weakens native plants that are dependent on, or flourish with, mycorrhizal fungi (Lawrence et al. 2003, Hopfensperger et al. 2011). In a meta-analysis of consequences of earthworm invasion in North American forests, Craven et al. (2016) found that cover of graminoids and exotic plants increased and cover of native plants of all functional groups decreased with increasing earthworm biomass.

Earthworms consume organic matter and incorporate it into deeper soil layers affecting carbon, phosphorus and nitrogen availability and flux (Rogers and Collins 2017). Changes in nutrient cycling, which vary by type of earthworm, can also affect plant establishment. Epigeic, or top soil worms, cause C and N transport and losses into soil A-horizons, possibly turning northern forests from C sinks into C sources, thus contributing to global warming (Bohlen et al. 2004, Cameron et al. 2015). Endogeic worms tend to mix mineral and organic soil layers. Anecic worms such as *Lumbricus terrestris*, a common species used for fishing bait, burrow vertically and have large burrows, leading to C and N transport into deeper B-horizons, with greater loss of organic C due to leaching (Crumsey et al. 2015).

Rare anecdotal reports of earthworms in Interior Alaska exist and specimens have been donated to the University of Alaska Museum, but very little was known about which species occur in Interior Alaska and no published records existed. Conventional wisdom of gardeners and long-time residents of Fairbanks is that the climate is too cold for earthworms. By combining standardised sampling with opportunistically donated museum specimens, this study documents for the first time the presence, identity and distribution of lumbricid earthworms in Interior Alaska. We hypothesised that earthworms would occur more often at higher elevations due to the common presence of permafrost-cooled soils in lower elevation valleys of Interior Alaska.

## Methods

**Figure 1.**
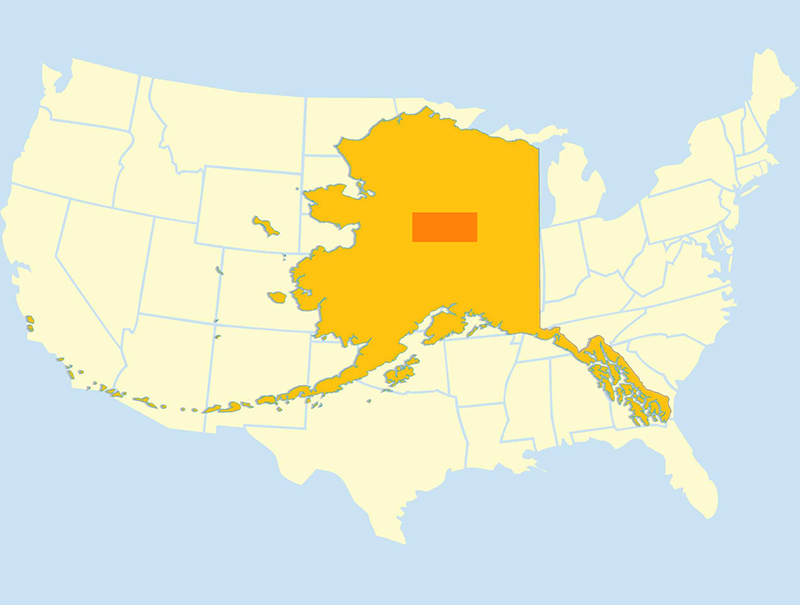
Map showing study region (dark orange rectangle) of Interior Alaska, centred around the city of Fairbanks, superimposed on map of the contiguous US states for scale. Original map by Laubenstein Ronald, U.S. Fish and Wildlife Service, is in the public domain.

We restricted the study area to a subset of Interior Alaska as defined by the map in Fig. 1. This bounding area (coordinates of region: 65.54001°N, -145.57928°W, 64.01478°N, -153.15545°W) was used to search the University of Alaska Museum Insect Collection's (UAM) online database, Arctos, for any Lumbricidae records based on citizen-donated specimens. Of the records identified, specimens collected in aquatic habitats and indoor vermiculture colonies were excluded so only feral, terrestrial Lumbricidae would be included in our study. The data for our voucher specimens, which are deposited in the University of Alaska Museum Insect Collection, can be accessed at the following link: https://arctos.database.museum/saved/interioraklumbricidae. This link is a live search, so any newly added Interior Alaska lumbricid records since 4 May 2018, will also be found.

Interior Alaska is dominated by boreal forest underlain with discontinuous permafrost and has a continental climate. The forest contains varying mixtures of conifers and deciduous trees including black spruce (*Picea mariana* (Mill.) Britton, Sterns & Poggenburg), which is abundant on permafrost soils in lowlands, white spruce (*Picea glauca* (Moench) Voss), Alaska paper birch (Betula pendula subsp. mandshurica (Regel) Ashburner & McAll.) and trembling aspen (*Populus tremuloides* Michx.), which are abundant on warmer, drier, uplands, amongst other tree and shrub species (Johnstone et al. 2010). Temperature extremes in Interior Alaska range from -94 to +95 °F (Hinzman et al. 2006), although Interior Alaska has warmed about 8.5°F during the winter between 1949 and 2016 (Alaska Climate Research Center 2018).

Since earthworms are thought to be most active during the spring and the fall (autumn) months (Gates 1961, Rogers and Collins 2017), we conducted standardised sampling during late summer and fall 2017. We selected locations across the Fairbanks vicinity in six relatively natural yet diverse areas (Table 1). Our standardised sampling locations were mostly in the College region of Fairbanks. Some sites were on the campus of the University of Alaska Fairbanks, which has been the site of an Agricultural Experiment Station since 1906. Elevations ranged over a span of 53 metres between the lowest and highest sites (#1 and #3).

We used a mustard extraction method (Lawrence and Bowers 2002, Reynolds and Wetzel 2008, Saltmarsh et al. 2016) and combined it with manual searching to increase our chances of finding worms (Sackett et al. 2012). A square, 50 cm × 50 cm quadrat was constructed by Barney Booysen with thin wooden boards to use as a guide when pouring the mustard-water mixture. To prepare this mixture, we mixed 2 oz. mustard powder with 1 gallon of water. At each site, we performed three replicate samples and pooled all collected worms. We cleared the top layers of debris, leaves, sticks, loose soil and root systems. We then took a photo of the quadrat and the surrounding plants and habitat and named the image file(s) according to the location and sample numbers. We then poured half of the mustard extraction mixture evenly across the ground in the quadrat. We waited 10 minutes before pouring the second half for the first few samples. However, after discovering the vast majority of worms (primarily enchytraeids) appeared within 5 minutes, we adjusted this wait time to 5 minutes between half gallons to enable more samples to be taken in less time. After pouring each half, we observed the ground within and around the quadrat closely and picked up any emerging worms with soft rounded forceps. Worms were immediately placed into 70% ethanol for killing and preservation. Once the third sample had been taken, a GPS reading was taken in the approximate middle of the three spots. The extent in metres was estimated and recorded along with the latitude and longitude, with the location name, site description, photograph IDs and the names of those who helped with fieldwork. We also recorded the time, weather, date of each location and notes on the success of the sampling. Most of this information was recorded in duplicate on a label that was placed inside the vial with the worms from all the samples at each location. The test of our elevation hypothesis was done using a combined dataset of UAM records and our standardised sampling.

We identified specimens in the UAM collection and those from our standardised sampling using the key in Reynolds (1977) and a Leica M165 C stereomicroscope at the University of Alaska Museum. Tissue samples from two specimens were submitted for DNA barcoding using LifeScanner kits. Statistical analyses were conducted using online QuickCalcs GraphPad software. For the elevation analysis, we used earthworm presence/absence grouped into bins spanning 50 m of elevation, rather than specimen counts, because the latter were not independent.

## Results

The standardised sampling yielded one earthworm specimen that appeared to be *Bimastos rubidus* from site #2 and eight specimens of *Dendrobaena octaedra* (Table 2) from site #6, both locations on the University of Alaska Fairbanks campus and of similar elevation (Table 1); no earthworms were found at the other four standardised sampling sites. The opportunistic (non-standardised) sampling yielded 28 earthworms of four species (Table 2).

Two specimen identifications were made using molecular data: those of *Aporrectodea caliginosa* and *Bimastos rubidus* (known as *Dendrodrilus rubidus* prior to Csuzdi et al. (2017)), which correspond with the following DNA barcode records on BOLD (Ratnasingham and Hebert 2007): (MOBIL6686-18) and (MOBIL6651-18) respectively. The latter species was also identified using the key in Reynolds (1977).

The COI sequence from our specimen of *A. caliginosa* from Fairbanks was 100% similar (*p*-dist) to sequences of *A. caliginosa* in BOLD BIN (Ratnasingham and Hebert 2013) BOLD:AAA2177, a clade widespread in the Palearctic and Nearctic based on specimen records in BOLD. Our sequence and other members of this BIN are members of the L3 lineage of *A. caliginosa* recognised by Porco et al. (2013) and Shekhovtsov et al. (2016).

**Figure 2.**
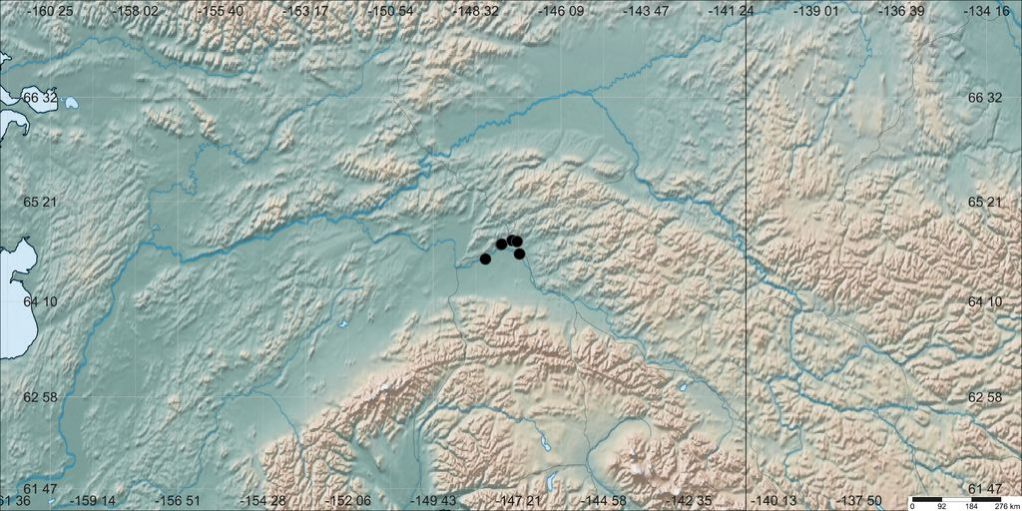
Map of earthworm record locations in Interior Alaska. Dark vertical line is the Alaskan-Canadian border. Map made using Shorthouse (2010).

Eight locality records (Fig. 2), of five lumbricid species (Table 2), are now known in Interior Alaska. Although the earliest of these records was collected in 2013, specimens remained unidentified until 2015.

**Figure 3.**
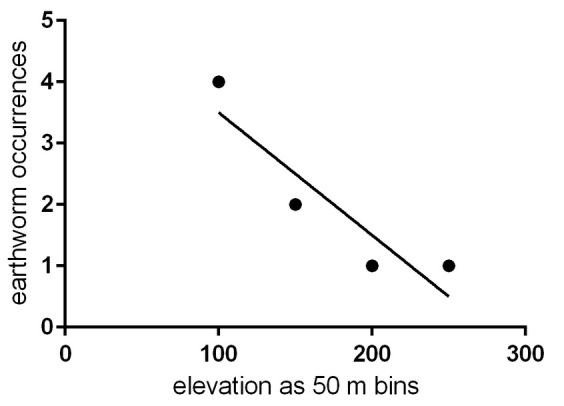
Elevation versus earthworm presence, grouped into bins spanning 50 m elevation. *R*^2^ = 0.8333, slope is not significantly different from zero (*p* = 0.087). Data are a combination of UAM records contributed by citizens and our standardised sampling data.

There was no significant relationship between elevation and earthworm presence when UAM data were combined with our standardised sampling data (R^2^ = 0.8333, *p* = 0.087, Fig. 3) although there was a slight tendency for earthworms to be less common at higher elevations. The standardised sampling data alone also showed no relationship with elevation (*p* = 0.845).

## Discussion

As a result of this study, five species of lumbricid earthworms have been identified as occurring in Interior Alaska. Four of these species were documented from opportunistic collections, with the standardised sampling adding one species, *Dendrobaena octaedra*. At least four of these species are European, or potentially Asian, introductions to North America (Reynolds 2015, Csuzdi et al. 2017). One species, *Bimastos rubidus*, known from the most sites (n=5), is thought to be native to North America (Schwert 1979, Csuzdi et al. 2017). All of these species had been previously documented from southeast and/or south-central Alaska (Costello et al. 2010, Reynolds 2016). Three of these four species (*Dendrobaena octaedra, Bimastos rubidus* and *Lumbricus terrestris*) are amongst the six lumbricid species known from Yukon Territory, Canada (Reynolds 2015), making it unsurprising that they also occur in Interior Alaska. The one species not known from Yukon Territory, *Eiseniella tetraedra*, is known from British Columbia, Manitoba and Nunavut Territory, Canada (Reynolds 2015), so its presence in Interior Alaska is also not surprising.

There is evidence that *Bimastos rubidus, Dendrobaena octaedra* and *Eiseniella tetraedra* are established in Interior Alaska, either due to numerous worms having been collected and/or observed at one site or one species having been collected at multiple sites. The *Lumbricus terrestris* and *Aporrectodea caliginosa* records from Interior Alaska are currently based on single specimens each from single sites, which we consider insufficient evidence to assume establishment.

We do not know when these species became established. Anecdotal reports of earthworms around Fairbanks suggest that some might date into the 1990s or earlier and, given there is evidence of *Bimastos rubidus* from >7,000 year old lake sediment in Ontario, Canada (Schwert 1979), at least this species may be native and thus have predated human establishment in Alaska. The Agricultural Experiment Station was established in 1906 and it is possible that exotic earthworms may have been in this region since then. However, reports from the public may be based on vermicomposting worms, which can spread from compost bins during the summer or based on enchytraeids, which can sometimes be confused with earthworms. Enchytraeids, also called 'pot worms,' are generally smaller and paler than earthworms, highly cold-adapted (Dash 1990) and widespread and native in Alaska. Alternatively, perhaps some or all of these species are recent introductions to the region, with persistence made more likely due to a warming climate. With increased warming, we expect additional populations and species of earthworms in Interior Alaska. Collection of mature specimens (with a clitellum) of suspected earthworms, preserved in 70-95% ethanol and donated to a public museum for identification and permanent archiving, is the best way to properly document populations of potentially exotic earthworms.

Our records of *L. terrestris* and *A. caliginosa* in Interior Alaska at 64.9°N latitude are apparently the northernmost records of these species in North America to date. In the Palearctic, *L. terrestris* has been collected at 69.7°N (GBIF.org 2018b) and *A. caliginosa* has been collected at 70.0°N (GBIF.org 2018a), both northernmost Palearctic records from Norway.

It should be noted that some confusion exists regarding the taxonomy of members of the *Aporrectodea caliginosa* species complex. Costello et al. (2010) reported *A. caliginosa* from southeast Alaska, but other records and check-lists from Alaska (Gates 1972, Reynolds and Wetzel 2008, Bowser 2010, Bowser 2015, Reynolds 2016) have used the name *Aporrectodea turgida* (Eisen, 1873), now considered a junior synonym of *Aporrectodea caliginosa* according to Blakemore (2008) and the Earthworm species searchable database (Csuzdi 2012), queried on 16 May 2018. See Pérez-Losada et al. (2009) for a history and discussion of taxonomy of this group.

The L3 lineage of *A. caliginosa*, to which our specimen belongs, is of European origin and appears to have become widespread relatively recently (Porco et al. 2013, Shekhovtsov et al. 2016).

We hypothesised that earthworms would be more likely to occur at higher elevations, away from permafrost valleys. There was no significant relationship between elevation and earthworm presence, although there was a tendency for worms to be more commonly found at lower elevations. However, with so few samples across an elevational gradient, it would be premature to draw firm conclusions. The greater number of earthworm records at lower elevations could simply be due to greater search effort spent at lower elevations.

We expected that earthworms would be more abundant in forested land than in developed or cultivated lands like fields and lawns, but this was not supported by our findings. The two sites that yielded earthworms in our standardised sampling were both grassy lawns. One had hard, compacted and rocky soil on a playing field on the UAF campus and the other had loose soil at the edge of a forest at the base of a hill on the UAF campus. None of the forested sites in our standardised sampling yielded earthworms, nor did other grassy sites. This suggests that, despite the favourable conditions in relatively undisturbed forest with higher moisture, loose soil, ample detritus, low traffic, lack of pesticides and shade, the grassy lawns may have been near where they were introduced. Worms may have been introduced to more disturbed areas due to landscaping or may be discarded fishing bait. This suggests the worms simply have not spread far beyond their original release sites.

However, the site at which *Eiseniella tetraedra* was collected is an early successional alder stand along the Tanana River, relatively far from human occupation (10.7 km downstream from a farm and 20.5 km downstream from the city of Fairbanks). Earthworms were observed in litter samples from this site in both summer 2016 and 2017 (personal observation RA). This parthenogenic species is known to disperse via flowing water (Terhivuo and Saura 2006). All other earthworm species records in this study were found less than 30 m from buildings or paved roads.

Knowing which exotic earthworm species are present, in addition to where they occur, provides important information on Alaska’s changing ecosystems, creates a present-day baseline with which to compare in the future and can help environmentalists determine if intervention and/or education needs to occur where human activity might be the leading cause of the spread of exotic earthworms. This study is a preliminary effort. We hope to expand our sampling efforts to better understand the earthworm fauna of Interior Alaska.

## Data Resources

The specimen data for the vouchers supporting the species presented in Fig. 2 and Table 2 can be accessed at https://arctos.database.museum/saved/interioraklumbricidae. The elevation data for Fig. 3 are in a supplementary file (Suppl. material 1.)

## Supplementary Material

Supplementary material 1 Data for elevation analysis Data type: occurrences in 50 m elevation bins
Brief description: Combination of standardised sampling and opportunistic sampling earthworm occurrence data for Interior Alaska.
File: oo_205518.csv Megan Booysen, Derek Sikes, Matthew Bowser, Robin Andrews

## Figures and Tables

**Table 1.** Interior Alaska sites sampled using mustard extraction.

**Site number**	**Site name**	**Latitude (°)**	**Longitude (°)**	**Habitat**	**Date**	**Elevation (m)**
1	Booysen home	64.82525	-147.903	permafrost ground	10-Sep-17	132
2	UAF campus	64.8511	-147.841	lawn edged with forest, side of road	14-Sep-17	142
3	UAF campus	64.86035	-147.837	forest near cemented trail	20-Sep-17	185
4	Sweeney and Mills home	64.8419	-147.851	lawn	21-Sep-17	134
5	West Valley HS	64.85091	-147.82	lawn near planted trees	2-Oct-17	132
6	UAF campus	64.85509	-147.835	playing field, grass	3-Oct-17	140

**Table 2.** Earthworm (Lumbricidae) records in Interior Alaska as of May 4, 2018. Year column indicates the earliest year of identification to species of Interior Alaska specimens; *n* indicates the number of Interior Alaska sites known for each species.

Species	Identified by	Year of Identification	*n*
*Aporrectodea caliginosa* (Savigny, 1826)	M. Bowser	2018	1
*Eiseniella tetraedra* (Savigny, 1826)	M. Bowser, M. Booysen	2016	1
*Dendrobaena octaedra* (Savigny, 1826)	M. Booysen, M. Bowser	2017	1
*Bimastos rubidus* (Savigny, 1826)	M. Bowser, M. Booysen, D. S. Sikes	2016	5
*Lumbricus terrestris* Linnaeus, 1758	D. S. Sikes, M. Booysen	2015	1
